# Foreign Hospital Intelligence

**Published:** 1894-04-07

**Authors:** 


### The Institutional Workshop.

### FOREIGN HOSPITAL INTELLIGENCE.

A New Hospital in Iceland.—There appears to be a distinct want for a hospital at Isafjord, Iceland, and endeavours are now being made to realise this plan. A committee has been at work for some time in order to collect the necessary funds, and the plan is being warmly recommended by several Danish doctors who have visited the locality.

Perambulatory Military Hospitals in Sweden.—The Swedish military authorities have asked for a Parliamentary vote of Kr. 714,000, or about £240,000, for perambulatory military hospitals. In asking for the vote reference is made to the exceedingly satisfactory results which were obtained by the German army during the Franco-Prussian war through the efficiency of their portable hospitals.

Although Copenhagen boasts a number of excellent hospitals, there has been quite a lack of hospital accommodation lately, more especially on account of the scarlet fever epidemic. There have also been several cases of small-pox, but of a regular epidemic there is no question so far. The appearance of this latter illness has, however, caused great inconvenience, as the hospital specially intended for small-pox and cholera patients had been used for scarlet fever patients, and it has been necessary to clear another hospital for the small-pox patients.

A New Air-cure Institution for Tuberculous Children.—An institution of this kind has quite recently been opened at Ormesson, close to Villiers, at the river Marne. It is a medico-pedagogic institute, where the principal treatment consists in an air, ozone, and microbicide cure. The new hospital, which is built on the most approved hygienic principle, comprises two two-storied garden villas, with electric light, &c. The one building contains one hundred beds, in addition to which there are tailor, shoemaker, basket maker, and other workshops, where the more intelligent of the children can be educated and trained. The other building is arranged after quite a new plan, and solely for the purpose of treating tuberculous children. It is situated at the other end of the garden, and is connected with the former building by a terrace and a covered walk. On the ground floor are the consulting and operation rooms, inhalation and bath rooms, and a bacteriological laboratory. On the terrace the children obtain their sun and air baths resting on comfortable couches. The interior of the other building consists of an immense hall, the arch roof of which is some twelve metres (forty feet) above the floor; it is fitted in two stories, with galleries four metres broad. This hall, which measures no less than 10,000 cubic metres, is intended for eighty children, so there is 120 cubic metres air for each child. The air is constantly renewed, passing away through an aperture in the roofing. In addition to this, an artificial atmosphere is let in morning and evening. This latter is produced by appliances in the cellar, where there, besides the air-heating apparatus, is a receptacle containing a solution of kerosene, eucalyptus, and turpentine, through which the warm air is led prior to its entering the hall. In addition, there are in the galleries, at the head end of the beds, leather bags which feed the hall with ozone.

British and American Hospital at Cannes.—At the end of last year a new law for the notification of infectious diseases came into force throughout France. British and American visitors to the Riviera who might be attacked by acute illness were thus liable to be removed by the authorities to the hospital of the municipality, where they must necessarily be far from comfortable. In the spring of 1893 a committee was appointed at Cannes to collect funds for the erection or provision of a building for the reception of English and American visitors and residents suffering from acute illness. This resulted in a subscription of £1,800, including £100 from the Hotel Keepers' Association at Cannes. The committee entered into an agreement with the Mayor of Cannes for the lease of Sunny Bank Villa for a term of years, with the option of purchase, and this has been fitted up for the reception of patients, and is now in full work under the management of Mrs. D. Norris, the Hon. Lady Superintendent, who has the assistance of an Edinburgh trained nurse. The necessity for a second building for the reception of acute cases other than infectious cases having become apparent, a further appeal has been issued, and meanwhile the Committee are erecting an isolation block. Sunny Bank is beautifully situated, the rooms devoted to patients are pleasant and home-like, and credit is due to Sir Sydney H. Waterlow, the treasurer, and to the English and American physicians and surgeons practising at Cannes, who have worked so hard to provide this most necessary institution for the protection and comfort of foreign visitors and residents who may be attacked by illness during their stay in the South of France. Sunny Bank was only opened at the end of last December. It may be regarded as a pay hospital, as the first-class patients pay 12 f. 50 c., and the second class 6 f. a day, including ordinary diet and nursing. The committee reserve the right of admitting free patients at their discretion.

